# Clinical Lecture on Club-Foot

**Published:** 1878-03

**Authors:** J. F. Miner


BUFFALO

### Medical and Surgical Journal.

VOL. XVII. MARCH, 1878. No. 8.

### Original Communications.

———:o:———

ART. I.—*Clinical Lecture on Club-foot.* By Prof. J. F. MINER, M. D. February 9th, 1878.

GENTLEMEN: A private patient consents to come before you for operation for club-foot, which consists in a peculiar distortion of the foot attended with a deviation from its natural direction, with change in the form of the bones, ligaments, tendons and muscles and usually with diminution in the strength, and sometimes in the length. It presents itself in many degrees, the deformity being sometimes so great as to render the individual an object of constant attention and remark, as well as greatly interfering with locomotion. On this account it is an affection which has always attracted the attention of medical men, inducing them to investigate its causes, and to devise means for its successful relief. True, club-foot is a congenital affection; it may, however, be imitated by causes operating after birth. It is generally believed that it may be acquired in true form, after birth, but such belief is probably unfounded; the bones are rarely if ever changed in form by any deviations from causes operating after birth. Though the foot is often displaced by accidental causes, such as constrained position until the soft parts,—ligaments and muscles,--are moulded into a new shape, and fixed in unnatural position, convulsions, irritation of the nerves, causing contraction of the muscles, various injuries, fractures, luxations, etc., etc; want of nervcus power often renders the muscles of one side of the foot lax and and yielding, and thus club-foot is acquired; but as I have said, true change in the form of the bones of the foot, arises from causes operating previous to birth.

Prof. Gross says that the etiology of club-foot has never been satisfactorily explained. "The hypothisis of arrested development, so warmly advocated by some modern pathologists, is altogether untenable, being essentially contrary to the facts in every particular. The imperfect growth, if any such really exist is not congential, as this doctrine teaches but acquired, being the result of causes which are brought to bear upon the child during its intra-uterine life, leading to shortening and contraction of certain muscles, and not to a want of development properly so-called. It must be acknowledged, however, that instances occasionally do occur, although rarely, which strongly favor the doctrine under consideration. Thus I have in my own practice, seen two infants born at the full term, but who died immediately after birth, who had well marked hare-lip, cleft-palate and club-foot, the result evidently, so far at least, as we can judge of such an occurrence, of an arrest of development." He says: "Arrest of development is untimable being contrary to facts in every particular, and then relates two cases which he thinks are evidently due to arrest of development." In this he is a little obscure or contradictory. Hare-lip and club-foot do not spring from the same causes even though associated in the same subject, at least there is a good way of accounting for non-union of the lip and palate in the centre, which does not apply to club-foot. If toes are left off from an otherwise perfect foot, it is certainly an arrest of development, but there is no arrest in club-foot, it is only mis-shapen. Every part of a natural foot is found in congenital club-foot, nothing is wanting, it is fully developed. Prof. Gross' two cases show nothing for arrested development, so far, at least, as club-foot is concerned.

He says again: "Another hypothisis of the formation of club-foot that has met with considerable notoriety, is, that the distortion is caused by the pressure of the uterus upon the feet of the infant during gestation in consequence of a deficiency of amniotic fluid. But the question may be asked, if such an effect may be produced upon the feet. Why should it not be exerted upon the hands, head, nose, chin, legs and knees! such a coincidence, suppose the doctrine to be true, ought to be of constant occurrence, yet it is so rare that it is probably not noticed in one case in a hundred of the affection. Besides it remains to be proved that women who have club-footed children have always a deficiency of amniotic fluid." He then goes on to say: "The most plausible view, perhaps, that can be framed in the present state of the science of the formation of club-foot, is, *that it is produced by a defect of nervous influence* leading to a permanent contraction in certain muscles and a corresponding retraction and incurvation of the bones into which these muscles are inserted."

It is very difficult to see any defect, or deficiency of nervous supply in congenital club-foot; the muscles, tendons and ligaments are well adapted to the deformed foot and require and receive the same amount of nervous supply that any foot requires, or receives; club-foot in all these respects is as perfect as any other foot, but is of bad shape. It is true that in some cases, the whole muscular apparatus of the leg suffers, is atrophied and weakened; but this may grow out of the fact, that the natural motions of the parts are interfered with, the organs are not at all adapted to natural use or motion, and become weakened and useless for the double reason of not being in shape for use, and not having muscles capable of motion. I have no ambition to disprove the existing theories concerning the causes of club foot, and have only noticed them to more forcibly impress you with the truth which I expect to demonstrate, that it really does depend upon pressure during intra-uterine life, from deficiency of amniotic fluid. (Specimens to illustrate here presented and described.) It appears remarkable that cause so adequate, so evident in some cases, and so manifestly liable to be in operation, should receive so little recognition, and still more remarkable that from so natural and philosophical an explanation physicians should wander off into occult and inconceivable influences of which they know nothing. It is not absolutely necessary that we determine positively the causes of this deformity, but it is well to entertain correct views in this respect, for upon our convictions as to its cause will depend, in some degree at least, the time and manner in which we shall attempt relief. On this account, mainly, I have indulged in quite a lengthy discussion which otherwise would possess very little practical value.

Club-foot presents itself under several varieties of form, of which there are four principal ones, differing from each other in the direction of the distortion, frequency of occurrence and details of treatment; but not in the general principles of treatment. These may be denominated, the *inverted, everted, extended* (phalangeal) *flexed,* or (calcaneal) varities, each name having reference to the manner in which the limb touches the ground. Thus in the inverted, the inner margin of the foot is turned upwards while in the everted it is turned downwards; in the phalangeal variety the heel is elevated while in the calcaneal it is depressed, the toes in the former being turned down, and up in the latter. Besides these general varities, there are several sub-divisions, depending upon a combination of two or more of the general divisions, as for instance the inverted and phalangeal, which is exceedingly common, and the inverted and calcaneal, which is much more rare. By far the most common form of club-foot is the inverted, usually called VARUS. The case now before you being of this form in which the patient walks upon the outer ankle, the great toe being directed inwards and upwards. The muscles of the calf and the adductors of the foot are contracted, hence there is elevation of the heel and a peculiar inward twist of the foot. This alteration occasions the most serious impediment to progression, and when it reaches the high degree here shown, gives a most disagreeable appearance to the limb. The outer margin of the foot, which in conjunction with the outer maleolus, chiefly sustains the weight of the body, is almost semi-circular in shape, rough and callous. The tendoschillis, is forced to the inner side of the foot, and forms a tense, rigid cord beneath the skin. When both feet are affected with *varus* they not unfrequently form an acute angle with the leg; they may approach so nearly to each other as to touch or even overlap.

The second variety, anciently called *valgus* may be described as the opposite of varus, the patient walking upon the inner side of the foot, while the outer side is drawn entirely from the ground.

The third or phalangeal variety, called by the older authors *equinus*, is attended by a shortening of the gastro-cremial and soleal muscles, aided sometimes, by the flexir of the toes. If any form of club-foot is acquired, this is most commonly the form assumed, and is said by writers to be "nearly always non-congenital."

In the fourth variety, the *calcaneal*, the limb rests upon the heel, the toes being drawn upwards towards the anterior surface of the leg. It is attended by a contraction of the anterior tibial muscle and of the extensor of the great toe, assisted sometimes by the common extensor of the foot. This variety is generally congenital, I have said, all varieties I believe are congenital in their real forms and all may be imitated in greater or less degree by causes operating after birth.

The changes which the bones, ligaments and muscles undergo, vary in the different varieties and in the differing degrees of the disease. The *tarsal* bones suffer the greatest alteration, being half dislocated, separated from each other and variously distorted. The calcaneum cuboid, scaphoid and astragulus are more deformed and changed in shape, but all the bones of the foot partake in the general malformation. The muscles are shortened on the one side and elongated and relaxed on the other; they are often misplaced. The ligaments do not at first, and in slight cases, appear much altered, while in those of long standing and severe form, they partake in the universal change.

It may here be remarked, that no one form appears alone, but that as they are combined in greater or less degree, so no description of minute changes can be made. It would answer no particular purpose to consider all the possible changes which the bones and muscles may undergo, as the *principles* of cure are the same in all cases, while the details vary with all the varying appearances which different cases present, each case requires an adaptation to itself.

TREATMENT. Club-foot should *always* receive early and efficient treatment, for the longer it is deferred, the more difficult it will be to effect a cure. This is true both of the congenital form, and the acquired or that induced by causes operating after birth. The bones at birth are exceedingly soft and yielding, and all the soft parts readily adapt themselves to any new position. At the very time of birth the sensation *is*, or *appears* to be less acute, and the deformed foot may be moulded back into natural form with wonderful facility. If rectified at this time the muscles sooner and more perfectly regain their natural functions, while the ligaments can hardly be said to have partaken of the deformity. In the worst grades of the deformity it is extremely difficult, if not impossible, when treatment has been neglected during the first years of life, to make a satisfactory cure. It can only be done by extensive division of tendons, and by wearing for a long time, retentive apparatus. The precise time at which the treatment should be commenced has been variously defined by different authorities. Some surgeons defer it two months some six and I am sorry to say some delay it for a year. It has been supposed that the newly born child cannot safely bear the operation and constraint and that all operative interference *must* be delayed until the child had attained to greater strength and vigor. In the slighter cases no division of tendons is necessary and but rarely is it necessary to do more than divide the tendo Achillis, even in the cases of greater deformity. All vigorous healthy children will bear this much of operation :—it can be made with perfect safety. It will not make an infant forsake the breast or appear in any way unwell. When you find that a newly born child has club-foot if the deviation is not great, grasp it firmly and resolutely and mould and twist it back—a little more than to its natural form, before you leave the house. Next morning repeat the operation, which you observe I complete for the present by applying adhesive plaster, so as to retain it fully round to its natural shape. If the tendo-schillis is too short, divide it sub-cutaneously and with but little parade, of course impressing the parents with the importance and necessity of it. Adhesive plaster is a favorite retentive apparatus with me, and I have never seen any other which answered for first dressing so fully the indications of treatment.

It requires some care and practice to use it satisfactorily but when you know how to apply it, you are in possession of the most simple, and above all, the most efficient means of retention ever yet adopted for the correction of club-foot. Other means may be used when the case has considerably progressed, but adhesive plaster is the best means of retention I have yet seen. I must not however fail to acknowledge the service rendered us in the treatment of this malady by the inventive genius of many of our mechanical friends, who have caused the manufacture of retentive apparatus of inestimable value in the latter treatment at least, and well calculated to be of service even immediately after operation. (Instruments shown and benefits explained.) I have never doubted but these machines will, if properly applied, retain the foot somewhat in natural form ; they are of great service to relieve the tedious after treatment, and on this account mainly they are urged upon your attention, but do not forget what I say about retaining the parts in place by adhesive plaster. Try the plaster and the boots, but if you are unsuccessful with either, do not blame either the boots or plaster, but remember that the fault may be altogether in the manner of application and in a lack of faithfulness and perseverence, either on your part or that of the friends, or nurse, though Plaster Paris bandage is now used a great deal for this purpose. It has advantages, but I have opportunity to show you my manner of early dressing, and it will be impossible for me to do much more, for every surgeon has his favorite methods, all differing in some respect but all alike in principle, and all productive of somewhat similar results.

Dr. Richard Barwell, of London, has recently expressed himself as strongly opposed to tenotomy as a remedy for club-foot. He has published a monograph and described an apparatus which, if properly applied, will generally, he thinks be sufficient to effect a speedy cure. His opinion is, that the tendo Achillis is almost the only tendon which should ever be divided. He recommends an apparatus, consisting of adhesive straps applied longitudinally and around the foot and leg. His object is to force the foot with *elastic dressings* into normal position, thinking thus to more highly favor muscular action. The division of the faulty tendons requires more care and attention than is usually supposed. To do it well requires skill, experience, judgment and a competent knowledge of the anatomy of the parts concerned. No preliminary treatment is required, and chloroform may be given or not. In infants anæsthetics are wholly unnecessary, but in children of six months or upwards it is better to use anæsthetics; local anæsthesia answers most admirably for this operation. *All* the faulty muscles may be divided at one time, where only club-foot is to be rectified; if the knee and thigh require division of tendons it might be well to divide the operation. The number and extent of the necessary divisions vary with each case; in talipes equinus, division of the tendo Achillis, is all that is required, while in inverted (*varus*) the tendon of the anterior tibial muscle, or of this and the long flexor of the toes, and as usually complicated with equinus the tendo Achillis may also require division. Age is no bar to tenotomy; the tendons will unite again, and you will have no anxiety about it. The reasons for operation in infancy have already been given, and are included in the softer and more pliable nature of the bones, ligaments and muscles and not from any objection to division of tendons later in life. Young adults are often benefitted and sometimes perfectly cured; so you must not understand me to say that *only* children can be cured, men at fifty have been reported as perfectly cured by operation.

The *Instrument* best suited to the division of tendons, is a narrow sharp pointed bistoury, now usually included in the common pocket-case, which makes only a mere prick through the skin; or an opening may be made through the skin and a very small, narrow, probe pointed bistoury introduced, and the division of the tendon effected by it. In either case the tendon to be divided is made tense when a slight sawing motion will effect a division of the tense cord, which is indicated by a distinct snap and giving way of the resistance. In division of the tendo Achillis it is well to pass the knife flat ways behind the tendon, between it and the deep parts, then by turning the edge against the tense cord, it will divide it to the exclusion of every thing else and it will give way with a distinct snap, and a separation of an inch in extent will often take place. This space will be filled with connective tissue similar to and taking the place of tendon. It has been proposed by Prof. Pancoast to divide the muscle higher up; to divide the soleal muscle in its lower portion, as admitting of more rapid union and rectification of deformity.

The posterior tibial muscle may be divided about an inch above the inner-ankle; the posterior tibial artery and nerve are the important structures necessary to avoid.

The most favorable situation to divide the tendon of the anterior tibial muscle, is in front of the ankle-joint, where it may be readily felt forming a distinct tense cord, care is necessary not to wound the anterior tibial artery. The *peroneal muscles* may most easily be divided a little above the outer ankle, as they run over the fibula. All contracted and unnaturally tense tendons may be divided in a similar manner, and the resistance having been thus overcome the foot can be placed in natural position. I have spoken of avoiding arteries and nerves; it seems to me that there is very little danger from this source. Tendons are made tense and other parts remain lax, the fibers thus tense are first divided and only from quite unnecessary division of parts can arteries or nerves be wounded. *If* any of the arteries are wounded, you are not to be disheartened, divide the artery completely across and apply pressure; this will generally be quite sufficient, of course it can be ligated if found necessary, but such a necessity can hardly arise. The absurd and useless method of delaying, after division of tendon for a few days, before bringing deformed members into shape has been wholly abandoned by intelligent surgeons. It was founded upon the error of thus obtaining less inflammation and less constitutional disturbance, but really produced much more, and made the second part of this operation, that is, the turning into place and dressing, much more painful than the first.

All deformities caused by, or attended by shortening of muscles, either in the upper or lower extremeties may be treated upon the same general principles. Your success in this class of surgical operations, as in all others, will depend upon the judgment and skill with which they are made, and I must not dismiss the subject without cautioning you not to promise yourselves, or your patients *too much.* You are dependent upon the assistance and co-operation, in most cases, of the parents or friends, which may *fail* upon trial. There are the usual sources of failure, and great intrinsic difficulties to be overcome.

